# Dr. Joseph Fowler

**Published:** 1909-02

**Authors:** 


### OBITUARY

---

Dr. Joseph Fowler, of Buffalo, died at his home in this city December 17, 1908, aged 61 years. He was a graduate of the University of Buffalo (1873), and had practised his profession in this city since that time. He had been ill for several months. He was a member of the Buffalo Academy of Medicine, of the Medical Society of the State of New York, for ten years a member of the Medical Staff of the Sister's of Charity Hospital, for twenty-two years Police Surgeon of Buffalo, and served for three years, from 1881 to 1884, as one of the County Coroners. Dr. Fowler took rank as one of the prominent physicians of Buffalo, was a public spirited citizen, and a man who commanded the respect of the community.

